# Recurrent bilateral chorioretinitis with positive Lyme serology: a case report

**DOI:** 10.1186/s13256-021-02804-7

**Published:** 2021-05-04

**Authors:** Reda Issa, Stephen A. M. DeSouza

**Affiliations:** 1 Associated Retina Consultants, 1750 E. Glendale Ave, Phoenix, AZ USA; 2 Department of Ophthalmology, University of Arizona College of Medicine Phoenix, Phoenix, AZ USA

**Keywords:** Lyme, Chorioretinitis, Retina, Case report

## Abstract

**Background:**

It has been disputed whether Lyme is a true causative agent in posterior uveitis or an incidental finding.

**Case presentation:**

This report presents a case of a 33-year-old Caucasian female with a remote history of Lyme disease who presented with blurry vision in the right eye. Exam and imaging revealed a right active chorioretinitis and positive Lyme serology. The patient was systemically treated with prednisone and antibiotics. Symptoms initially improved, but she later developed a localized choriocapillaritis in the left eye. Steroids and antibiotics were restarted many times with fluctuating course of the disease. The patient was then started on chronic steroid-sparing immunosuppression, which has controlled the condition without recurrence.

**Conclusions:**

The current report presents a unique case of recurrent bilateral chorioretinitis with positive Lyme serology and raises the question of the existence of true Lyme-associated uveitis.

## Background

Lyme disease is a tick-borne infection caused, most commonly, by the *Borrelia burgdorferi* spirochete and involving multiple organs, including the eye. The disease affects multiple organs, including the classic skin lesions (erythema chronicum migrans), arthritis, neurological manifestations (meningitis and peripheral facial palsy), and even cardiac involvement [1]. Lyme disease has nonspecific symptoms in the eye, with findings ranging from conjunctivitis and keratitis early on to various forms of uveitis, neuroretinitis, retinal vasculitis, and cranial nerve palsies in later stages of the disease [2, 3]. There are many unknowns in cases of possible Lyme-associated ocular inflammation, including questions of testing and treatment, and whether Lyme truly causes the uveitis or is an incidental finding. This presentation adds another data point in the world literature trying to answer those questions.

In this case report, we describe a case of recurrent bilateral chorioretinitis with positive Lyme serology.

## Case report

**Fig. 1:**
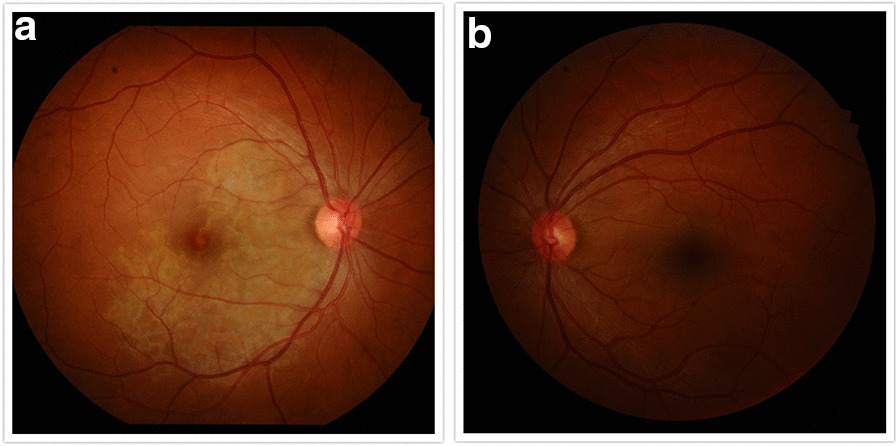
**a** Funduscopic examination of the right eye showing diffuse subretinal pigmentary changes involving and inferonasal to the fovea. There were some small (~ 200 μm) areas of active choroiditis temporal to the fovea. There is no vitritis. **b** Normal fundus photograph of the left eye

A 33-year-old Caucasian female patient was referred to our practice for evaluation of central blurred vision in the right eye for 2–3 weeks. She denied any pain. She was a healthy woman with no ocular history. Her only prior medical history included an episode of bilateral knee arthritis in her late teens, diagnosed as Lyme disease and reportedly treated with oral antibiotics. She had a 5-month-old baby and was breastfeeding. This was her third pregnancy. She had no known visual problems after her initial Lyme episode. Family history included a mother with cancer and father with glaucoma. She was a non-smoker and denied any drug use. She occasionally consumed alcohol. She was not taking any medications. Visual acuity was 20/50+2 in the right eye and 20/20– in the left eye. Slit lamp examination was unremarkable with a quiet anterior chamber. Funduscopic examination of the right eye (Figure 1a) showed diffuse subretinal pigmentary changes involving the fovea, without any vitritis. There were some small (~ 200 μm) areas of active choroiditis temporal to the fovea. Fundus examination of the left eye was unremarkable (Figure 1b).

**Fig. 2:**
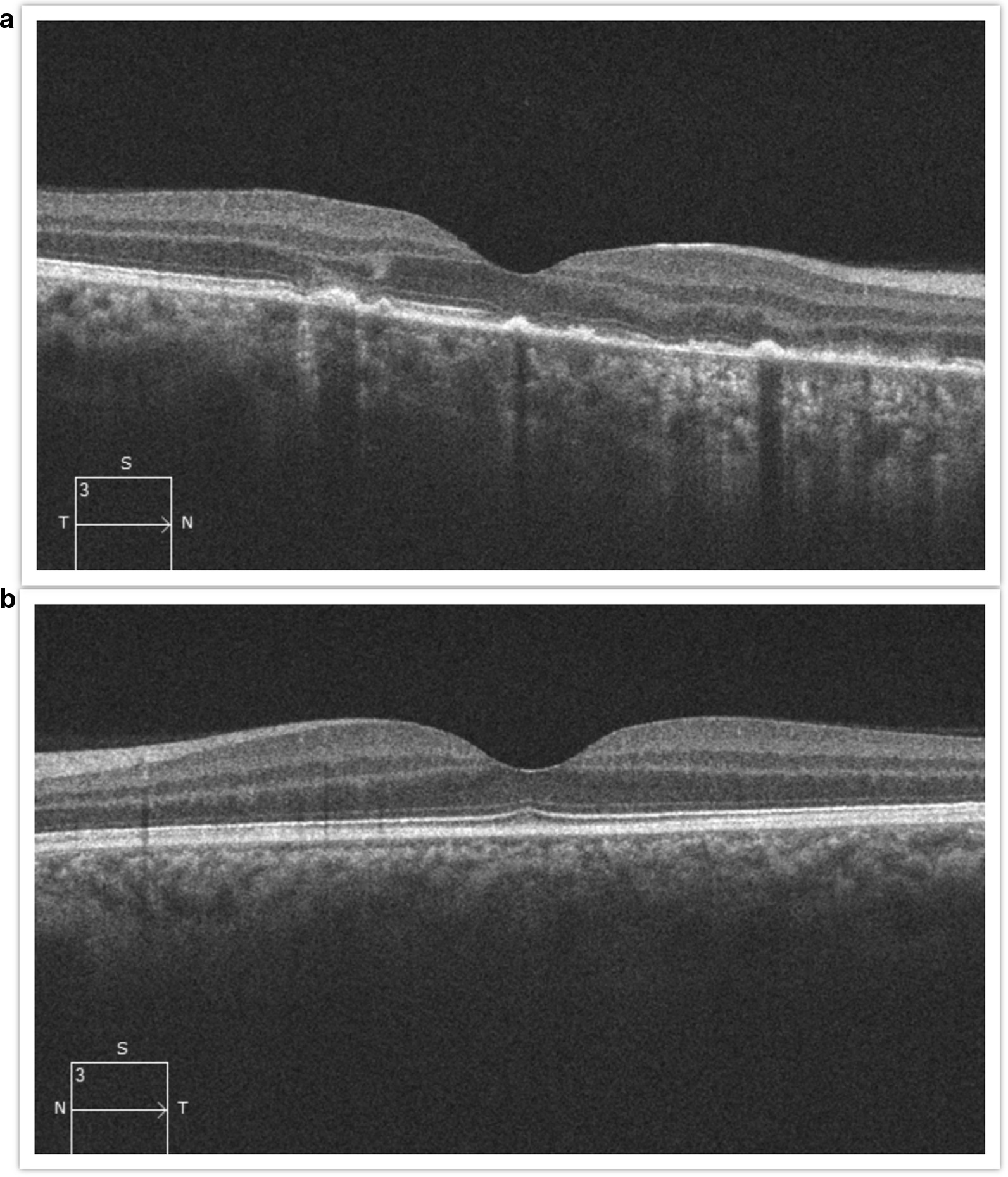
Optical coherence tomography showing retinal pigment elevation with thinning and disruption of the ellipsoid layer in the right eye (**a**), and a normal foveal architecture in the left eye (**b**)

**Fig. 3:**
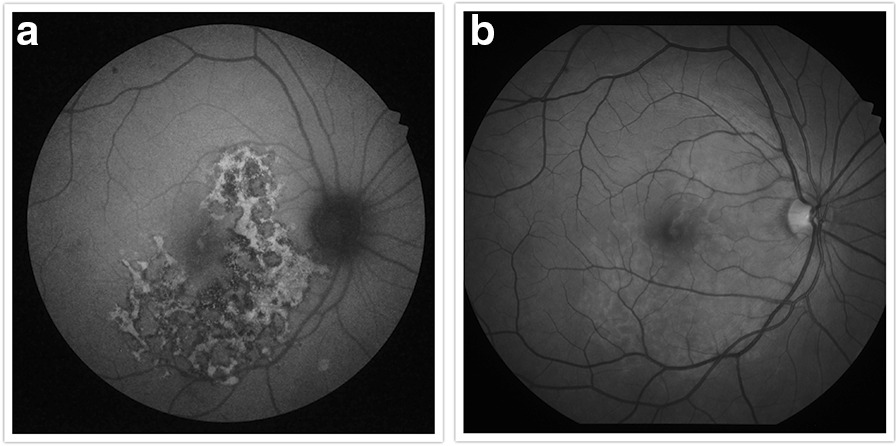
**a** Fundus autofluorescence of the right eye showing a leading edge of increased autofluorescence. **b** Red-free fundus photograph of the right eye highlighting the lesions seen clinically

**Fig. 4:**
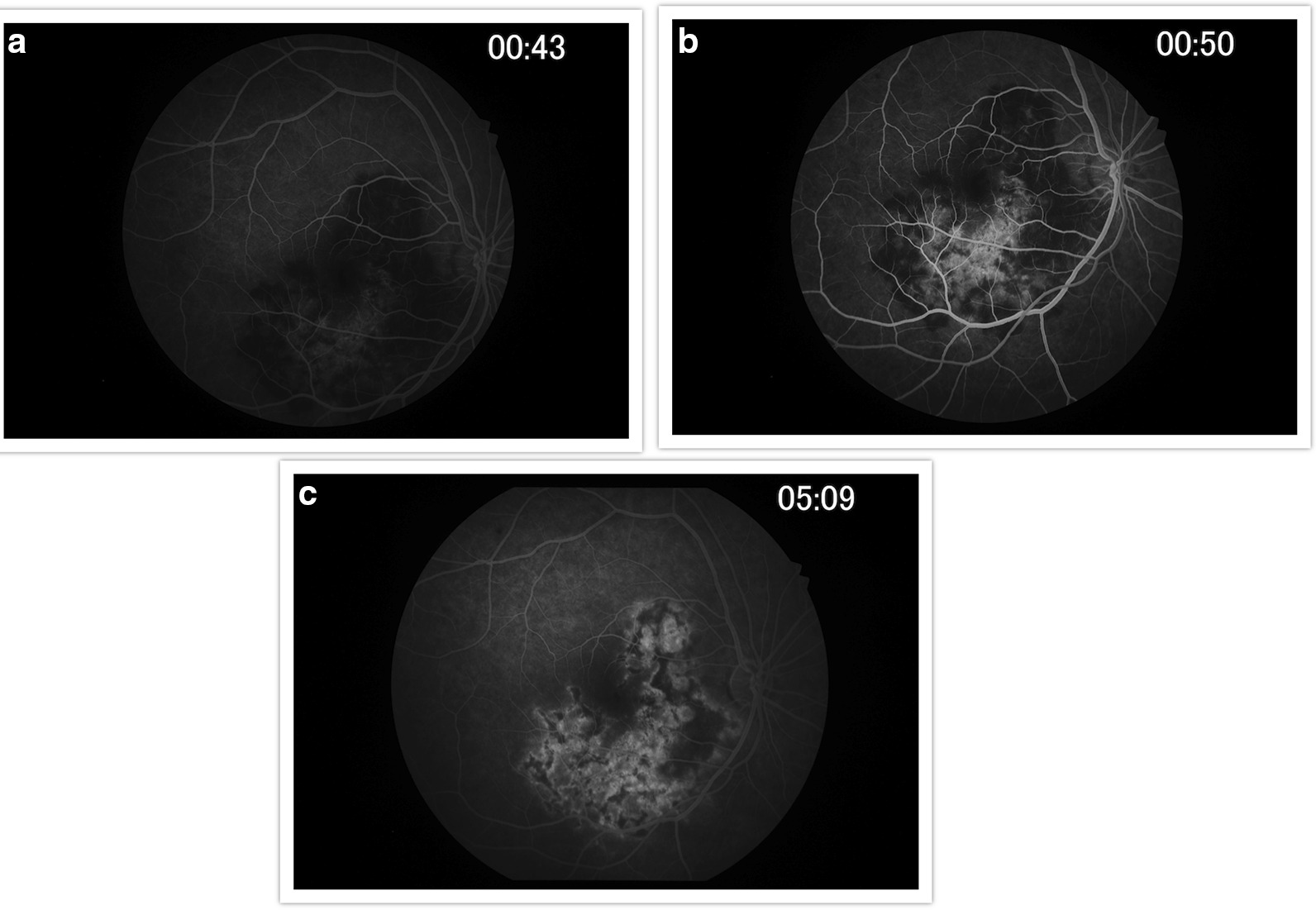
Early (**a**), laminar (**b**), and late (**c**) phase of intravenous fluorescein angiography of the right eye. There is initial blockage followed by late staining of the diffuse area of chorioretinal pigmentary changes seen clinically

**Fig. 5:**
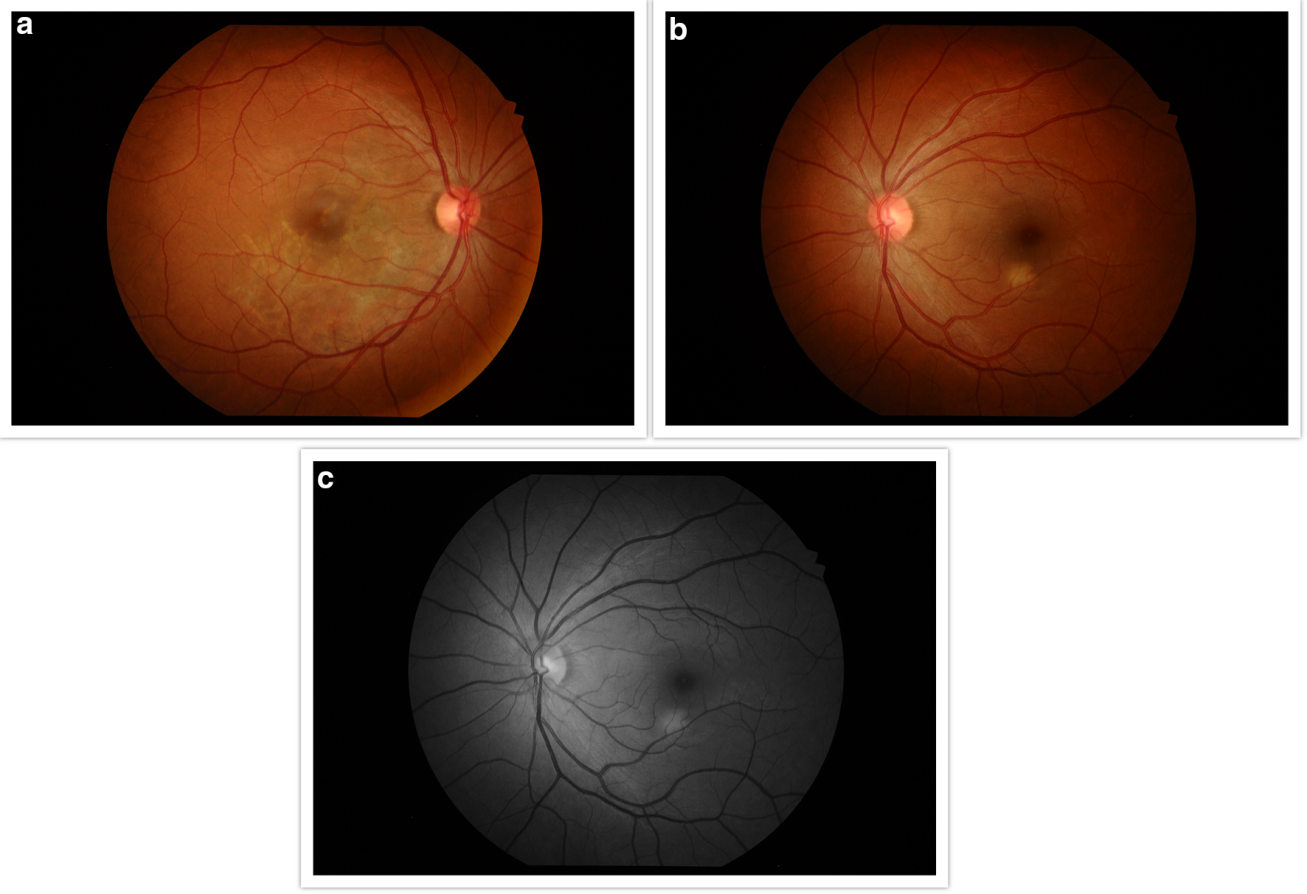
Fundus photograph of the right eye **a** 4 months after presentation showing a stable and chronic lesion. The left eye **b**, **c** shows a new white lesion inferior to the fovea

**Fig. 6:**
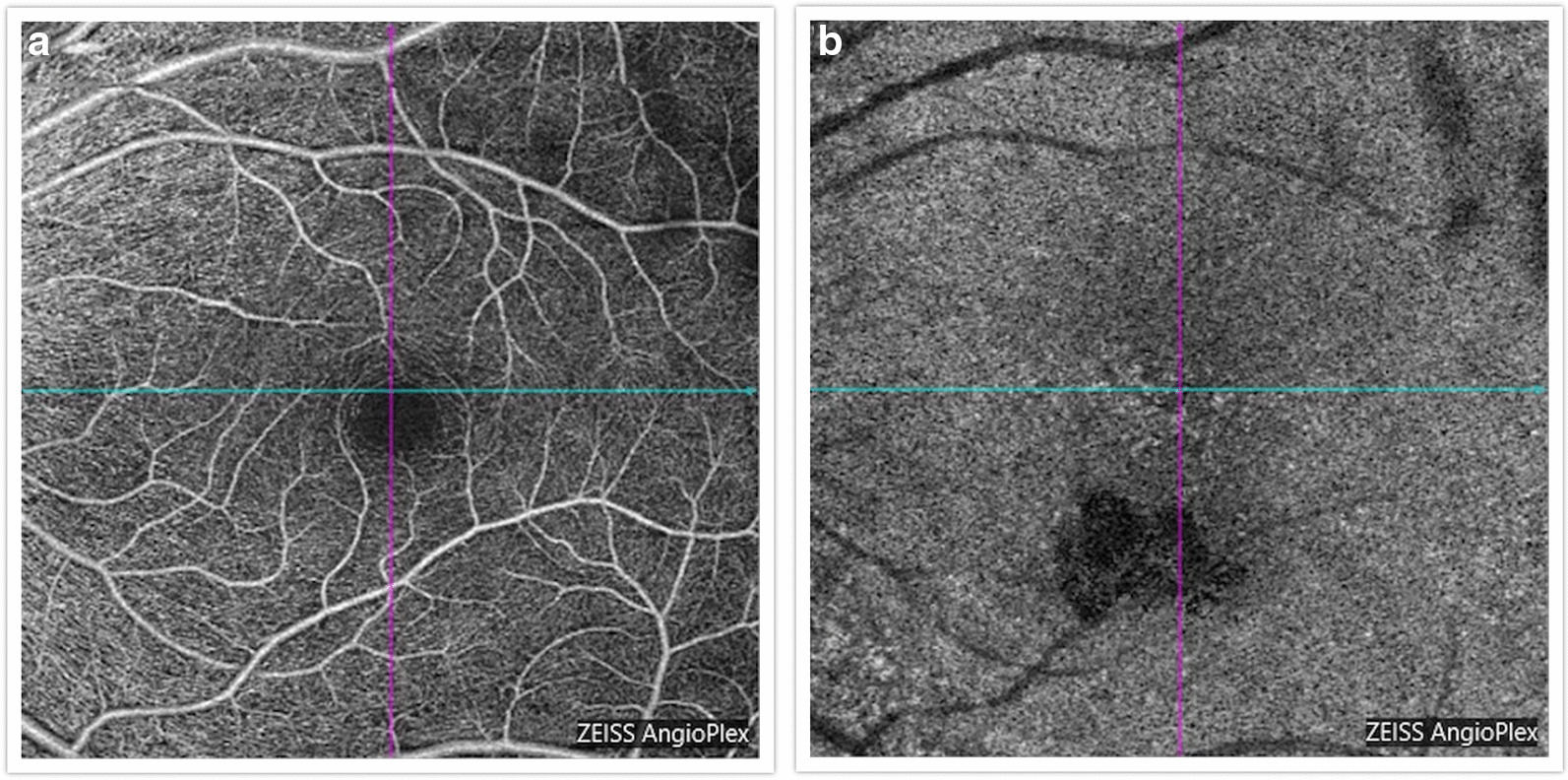
OCTA (**a** en face superficial, **b** en face choriocapillaris) showing an outer retinal hypoperfused area

**Fig. 7:**
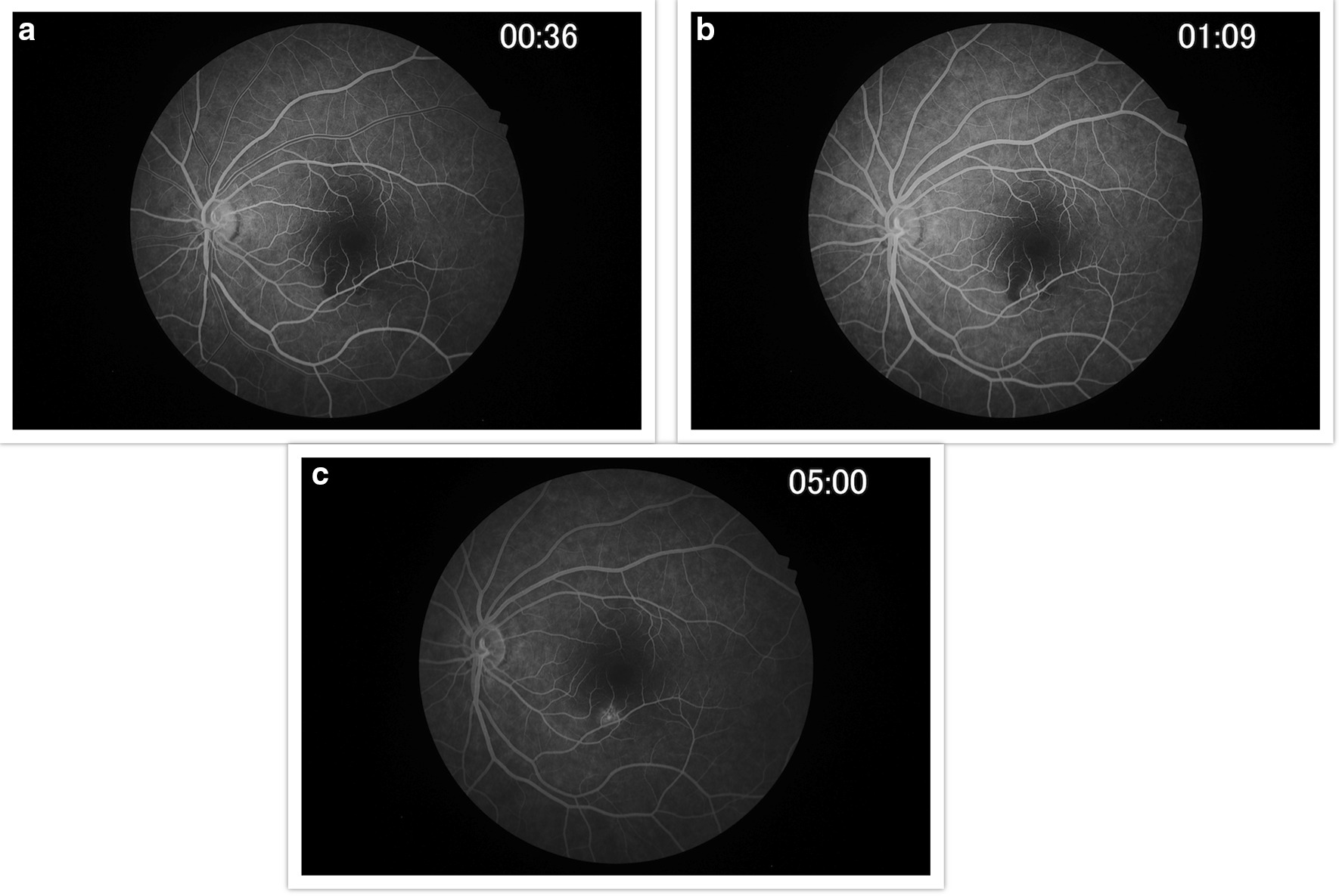
**a**–**c** Fluorescein angiography of the left eye 4 months after initial presentation showing early hypofluorescence of the lesion inferior to the fovea followed by late staining with possible central leakage

**Fig. 8:**
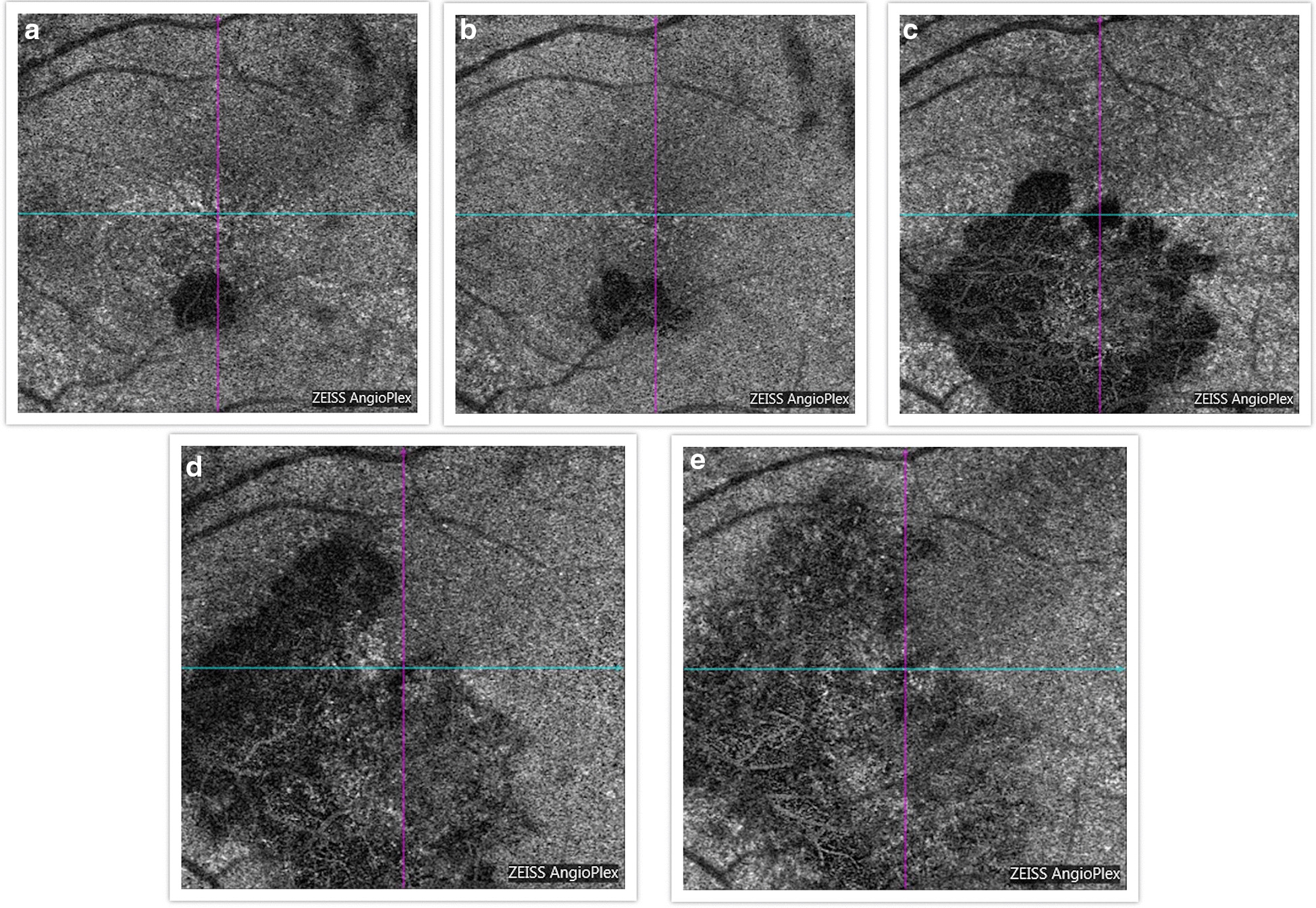
**a** OCTA (level of choriocapillaris) at presentation of new left eye symptoms. **b** OCTA 1 week later. **c** OCTA 2 months later showing an enlarged area of choriocapillaritis (at which points high-dose steroids were restarted). **d** OCTA 2 weeks later shows further progression of the lesion (at which point prednisone was increased to 60 mg and doxycycline restarted). **e** OCTA 2 months later shows a slightly increased size of the lesion but appearing inactive

**Fig. 9:**
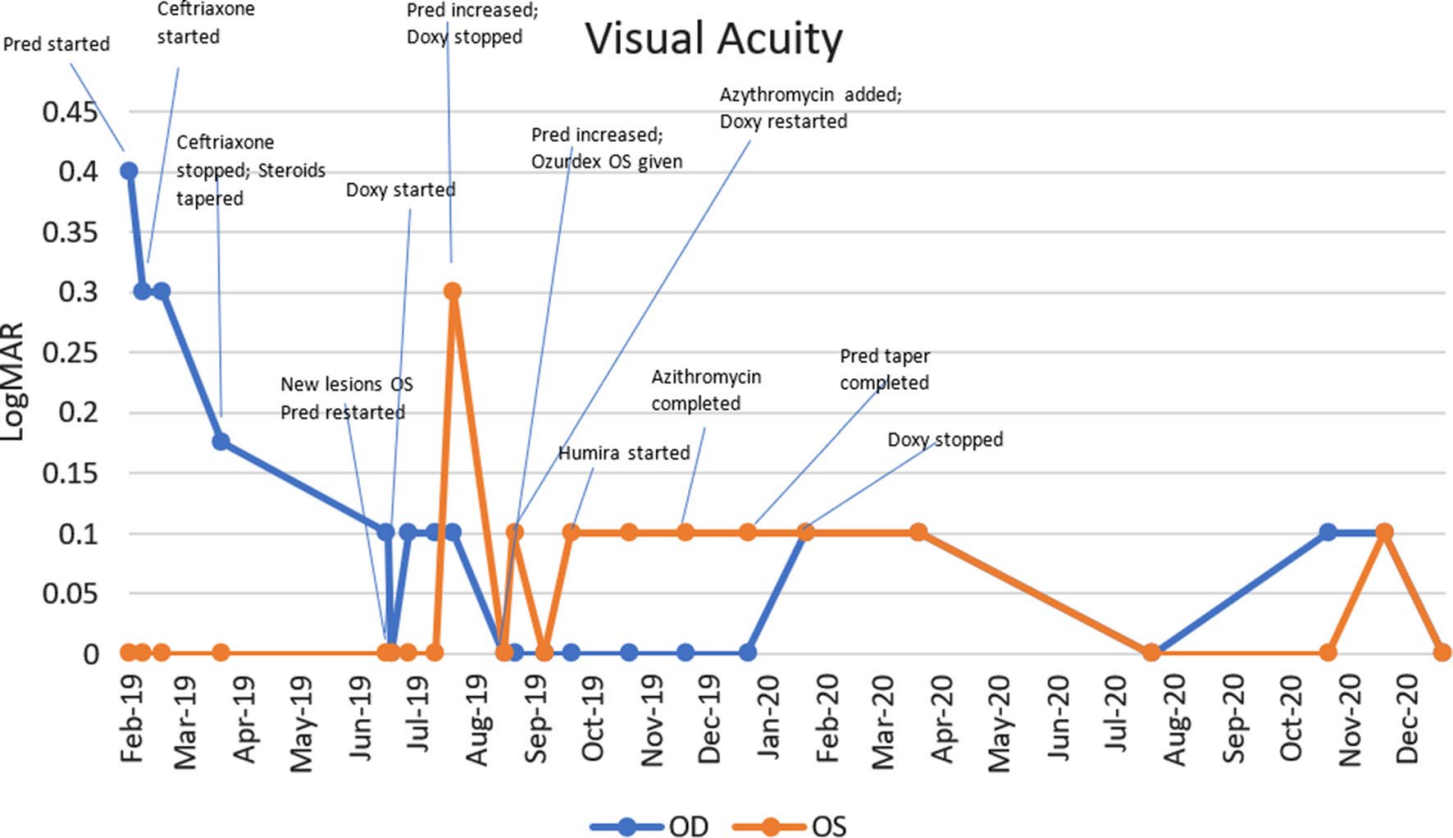
Timeline of visual acuity and interventions since presentation

Findings were confirmed by spectral domain optical coherence tomography (OCT; Carl Zeiss Meditec, Dublin, CA), which showed retinal pigment elevation with thinning and disruption of the ellipsoid layer in the right eye (Figure 2), and normal foveal architecture in the left eye. Fundus photography, red-free, autofluorescence, and intravenous fluorescein angiography (IVFA; Kowa, Aichi, Japan) was performed, showing a leading edge of increased autofluorescence with initial blockage followed by late staining (Figures 3 and 4). She was started on 50 mg oral prednisone. A thorough laboratory workup, including Lyme, rapid plasma reagin (RPR), fluorescent treponemal antibody absorption (FTA-ABS), antinuclear antibody (ANA), angiotensin-converting enzyme (ACE), lysozyme, and antineutrophil cytoplasmic antibodies c-Antineutrophil Cytoplasmic Antibodies (ANCA) and p-ANCA, was negative. Her complete blood count was normal with a white cell count of 10.9, a hemoglobin level of 13.7, and hematocrit of 40.6. Her comprehensive metabolic panel including liver function tests was normal. Her creatinine was 0.6. A thorough laboratory workup was ordered and was negative except for five bands of immunoglobulin G (IgG) for Lyme and two bands of immunoglobulin M (IgM) for Lyme [4, 5]. The patient admitted to recently traveling to Maine, known for its high rate of Lyme disease according to the Centers for Disease Control and Prevention (CDC), and hiking extensively, but without a known tick bite. The infectious disease service was consulted and confirmed the diagnosis of suspected active Lyme disease. The patient was admitted to the hospital and received intravenous ceftriaxone 2 g twice daily for 4 weeks. At admission, her blood pressure was 106/69 mmHg, pulse 70 beats/minute, and temperature 97.4 °F. At the end of February 2019, her visual acuity improved to 20/40 and prednisone was decreased to 40 mg. In March 2019, her prednisone was decreased to 30 mg and she completed 4 weeks of ceftriaxone. Her prednisone was further tapered, to 20 mg for 2 weeks, then 10 mg for 1 week, then 10 mg every other day for 1 week, then stopped. In June 2019, her vision returned to 20/20. At that visit, a new lesion was noted in the left eye (Figure 5). Optical coherence tomography angiography (OCTA) (Figure 6) and IVFA (Figure 7) revealed a hypoperfused outer retinal lesion in that area. She was restarted on 50 mg oral prednisone for 3 days with a taper by 10 mg every 3 days. Repeat Lyme testing was conducted, showing three positive IgG bands and two positive IgM bands. After repeat consultation with the infectious disease service, the patient was started on 100 mg oral doxycycline twice daily. In August 2019, after finishing her doxycycline and tapering off her prednisone, she was noted to have progression of the subretinal lesion in the left eye and decline in vision. She was restarted on 50 mg oral prednisone and sent to rheumatology for consideration of steroid-sparing immunosuppression. Figure 8 shows the progression of the OCTA, at the level of the choriocapillaris, over this period. The patient then received a dexamethasone intravitreal implant 0.7 mg (Ozurdex, Allergan, NJ) toward the end of August 2019 and was kept on high-dose prednisone (increased to 60 mg). Azithromycin 500 mg oral daily was also added, and doxycycline 100 mg oral twice daily was restarted. Rheumatology started the patient on adalimumab (Humira, Abbvie, IL) in September 2019, and then steroids were slowly tapered (by 5 mg every week). Repeat Lyme titers revealed four positive IgG bands and two positive IgM bands. She finished her azithromycin course in November 2019. In December 2019, she finished her prednisone taper, and in January 2020, she was taken off doxycycline and kept on adalimumab only. There has been no further recurrence of her findings or of her symptoms, and her vision remained between 20/20 and 20/25 for over a year. Figure 9 shows a timeline of her visual acuity since presentation (no known prior visual acuities but reportedly normal).

## Discussion

This presentation shows an interesting case of recurrent bilateral chorioretinitis in a patient with a history of Lyme disease and positive Lyme serology, and adds another data point to the literature about the possible involvement of Lyme in retinal conditions while highlighting the difficulty in establishing causality.

Lyme disease is a tick-borne disease most commonly caused by the spirochete *Borrelia burgdorferi*. Eye involvement is rare, affecting less than 1% of Lyme cases and representing about 1% of all cases of uveitis [1]. The seroprevalence of *B. burgdorferi* among a large case series of uveitis patients from France was 7.9%, similar to the general population, the majority of whom had other etiologies to explain their uveitis, with only 1.6% found to be true Lyme uveitis cases (with history of tick bites and resistance to steroid-only treatment). Another large case series reported a rate of 4.4% [6]. For these reasons, it is not advised to screen all uveitis patients for Lyme disease as the seropositivity can be incidental. Of important note, IgM and IgG may persist for years without reactivation of borreliosis [7]. Lyme-associated uveitis has a very varied presentation: from anterior, to intermediate, to posterior uveitis [8]. It can be unilateral or bilateral, granulomatous or not, and with or without vasculitis. The main common feature is a history or risk of possible exposure to Lyme and extraophthalmic manifestations. The principal symptom is decreased vision in Lyme uveitis patients.

Lyme disease can even manifest as white dot syndrome showing multifocal white dots in the posterior pole in addition to the more common findings such as retinal vasculitis and anterior or posterior chamber inflammation. Patients with Lyme disease and severe ocular involvement should be treated with systemic antibiotics and steroids (+/− topical steroids) [9]. A case of Lyme disease with a serous retinal detachment and chorioretinal folds was shown to be responsive to antibiotics alone [10]. At least two cases were reported of patients with acute posterior multifocal placoid pigment epitheliopathy (APMPPE) who had an acute Lyme infection, with blurry vision and all signs and symptoms resolving upon successful treatment of Lyme disease with combination steroids and antibiotics [11, 12]. Nevertheless, a definite causational relationship between Lyme and APMPPE has not been established in the literature.  Those papers and a few other case reports do suggest a possible association, but a large case series of 18 patients with APMPPE [13] showed that none of the patients had borreliosis. However, antiborrelia antibody concentrations decline to even undetectable levels in patients with culture or polymerase chain reaction (PCR)-proven Lyme [5], potentially explaining the negative results in that case series. All this literature shows inconclusive evidence of the true causality of Lyme disease in posterior uveitis. Our patient had some features consistent with APMPEE, such as the duration of less than 6 months and mostly posterior pole lesions, and some features consistent with relentless placoid chorioretinitis, mainly the recurrent episodic nature requiring immunosuppression for control.

Evidence of our patient’s hypofluorescence and hypoperfusion in the outer retinal layers of the left eye on fluorescein angiography and OCT angiography, respectively, consistent with localized choriocapillaritis, was also demonstrated in a report of three patients with a primary inflammatory choriocapillaropathy due to Lyme *Borrelia* [14]. This has been posited as being the result of immune complex deposition, in response to disseminated spirochetes, at the level of the choriocapillaris, possibly similar to what occurs in acute syphilitic posterior placoid choroiditis [12]. Recently, a case from Turkey was published showing unilateral chorioretinitis, similar in presentation to our patient, with western blot verified Lyme disease, who had significant improvement following treatment with doxycycline and 10 days of oral prednisone, without recurrence [15]. Of note, a literature review has not returned a specific association between pregnancy and activation or contraction of Lyme disease [16, 17]. Additionally, literature has shown that Lyme infection during pregnancy is benign to the fetus with no causal teratogenicity confirmed, despite earlier suggestive reports [16, 17]. However, our patient's symptoms only began after her infant was about 2 months old.

## Conclusions

Our report shows a unique patient who has what appears to be a case of recurrent bilateral chorioretinitis but with persistent IgG and IgM serology for Lyme disease. This case highlights the uncertainty Lyme serology creates and the difficulty in ascertaining a causal relationship.

## Data Availability

Not applicable.
